# Spreadable Magnetic Soft Robots with On-Demand Hardening

**DOI:** 10.34133/research.0262

**Published:** 2023-11-29

**Authors:** Zichen Xu, Yuanhe Chen, Qingsong Xu

**Affiliations:** Department of Electromechanical Engineering, Faculty of Science and Technology, University of Macau, Macau, China.

## Abstract

Magnetically actuated mobile robots demonstrate attractive advantages in various medical applications due to their wireless and programmable executions with tiny sizes. Confronted with complex application scenarios, however, it requires more flexible and adaptive deployment and utilization methods to fully exploit the functionalities brought by magnetic robots. Herein, we report a design and utilization strategy of magnetic soft robots using a mixture of magnetic particles and non-Newtonian fluidic soft materials to produce programmable, hardened, adhesive, reconfigurable soft robots. For deployment, their ultrasoft structure and adhesion enable them to be spread on various surfaces, achieving magnetic actuation empowerment. The reported technology can potentially improve the functionality of robotic end-effectors and functional surfaces. Experimental results demonstrate that the proposed robots could help to grasp and actuate objects 300 times heavier than their weight. Furthermore, it is the first time we have enhanced the stiffness of mechanical structures for these soft materials by on-demand programmable hardening, enabling the robots to maximize force outputs. These findings offer a promising path to understanding, designing, and leveraging magnetic robots for more powerful applications.

## Introduction

Wireless-actuated magnetic soft robots can reach deep tissue and corners within the human body, and their real-time navigation inside the human body plays an important role in the precise treatment of patients in the biomedical field [1–6]. Furthermore, ultrasoft structures help these robots work in confined spaces and crowded corners, avoiding potential blockage and physical injuries [7–10]. Researchers have introduced soft functional materials to diverse robot designs, ranging from responsive hydrogels [11–14] to well-designed elastomer materials [7,15,16], demonstrating intriguing performances in biological compatibility, chemical reactions, and medical treatment. However, for actual clinical applications, there remain several limitations in medical assignments in vivo when only relying on magnetic miniature robots, owing to the functionalities and self-performance parameters such as output force and structure stiffness. These inherent disadvantages prevent further medical development and research, requiring more flexible and adaptive deployment and utilization strategies. It remains a challenge to enable these miniature magnetic robots to finish complex assignments by themselves, which is over-forward-looking and impractical for current research [17,18]. The synergistic development of magnetic tiny robots with existing medical devices is promising and practical to introduce these new findings into clinical applications as quickly as possible [19,20].

Magnetic miniature robots are useful to be coupled with plenty of traditional medical equipment, which brings great performance enhancement [5,19–22]. Magnetic actuation empowerments introduce extra functionalities, including navigation and execution, and enable brand-new utilization strategies for several professional instruments, such as magnetic catheters [23,24]. How to combine magnetic miniature robots with traditional medical equipment efficiently is worthy of research. Currently, 2 primary schemes have been proposed and implemented. One is introducing magnetic elements to related equipment fabrication [5,19,23], and the other is using adhesion to deploy magnetic robots on related equipment [25]. Although both provide exciting insights into achieving great performance improvement, they failed to fully exploit and take magnetic robots’ advantages, namely, untethered robotic properties. Concerning the former, the magnetic elements cannot be separated from the equipment body to work as a single magnetic miniature robot, which undoubtedly will influence its further execution in confined spaces. Regarding the latter, although magnetic robots can achieve reprogramming and disintegrating [25], they failed to present sufficient contributions to medical uses after leaving the main body. In addition, normal magnetic robot adhesion does not provide more functional enhancements beyond magnetic actuation. These annoying issues spur the development of initial magnetic robot designs and the deployment strategy so those robots can perform well in coordination with existing equipment.

In addition, it needs outstanding adaptivity and deformability to enable magnetic robots to work flexibly with current medical tools, which means ultrasoft structures, reconfiguration, and exceptional adhesion. The demanding requirements essentially limit their prospects since it is challenging to achieve robotic properties with sufficient stiffness and force outputs by such ultrasoft structures [10,26,27]. To tackle this issue, one choice is to utilize liquid metals that undergo liquid–solid phase transitions [28,29]. Such an approach exploits the melting and cooling phase transition to realize different robot forms for various assignments. However, the phase change procedure relies on a temperature increase or reduction, which takes certain time and is potentially dangerous due to the temperature changes in vivo. Seral mechanically triggered materials with tunable stiffness provide excellent performance benchmarks, demonstrating swift stiffness enhancement [30]. Nevertheless, they have not been widely used in further applications, especially from the perspective of robotics, where the induced enhancement of stiffness has not been well presented, such as potential improvement in output forces. More practical magnetic miniature soft robots are needed to facilitate healthcare services, and they must have programmable dynamic stiffness to cope with diverse demands swiftly. Robots should also exhibit powerful output forces and flexible deployments, which have seldom been implemented.

**Fig. 1.**
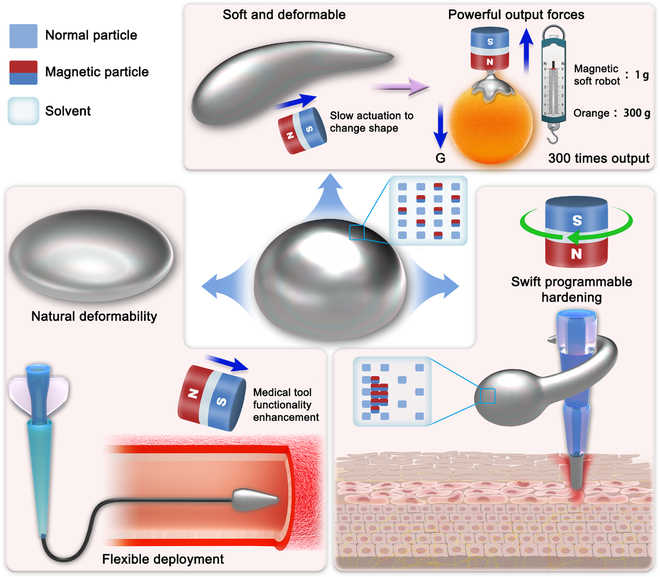
Illustration of the proposed magnetic miniature soft robot for diverse applications.

Herein, we report a design strategy that utilizes a mixture of magnetic particles and non-Newtonian fluidic soft materials to produce programmable, hardened, adhesive, and reconfigurable soft robots (Fig. 1). By applying external magnetic force stimulus, its physical properties, including sizes, shape, adhesion, and stiffness, can be programmed on-demand in real time. The fluidic properties and adhesion enable the proposed soft robots to be stably, flexibly adapted, and deployed to most surfaces with various sizes and shapes, facilitating efficiency promotion while working with medical devices. The introduction of non-Newtonian fluid material enables multimodal on-demand hardening for different assignments. A rapidly oscillating external magnetic field stimulus is applied to swiftly harden the soft material, activating its non-Newtonian fluid properties to resist intense interactions [31,32]. A static, strong magnetic field (100-mT level) can also organize the magnetic particles inside the soft robots to resist external forces. These findings bring a great enhancement in robot performance. In our experiments, the proposed robots can even grasp and actuate objects 300 times heavier than their weight under the actuation of magnets (height: 30 mm, diameter: 30 mm). Overall, our contribution can be summarized as follows: (a) Utilizing ultrasoft and adhesive properties to deploy soft magnetic robots widely and flexibly, thus increasing their potential applications; (b) designing programmable hardened soft materials and related magnetic miniature robots; and (c) enabling miniature magnetic robots to provide large output forces. This study paves a new path to designing and utilizing magnetic soft robots. Adding magnetic elements to functional materials is a novel paradigm that promises to fully exploit the properties of responsive materials to improve the performance of magnetic robots and promote their use in related medical applications.

## Results

### Magnetic soft robot demonstration and adhesion

**Fig. 2.**
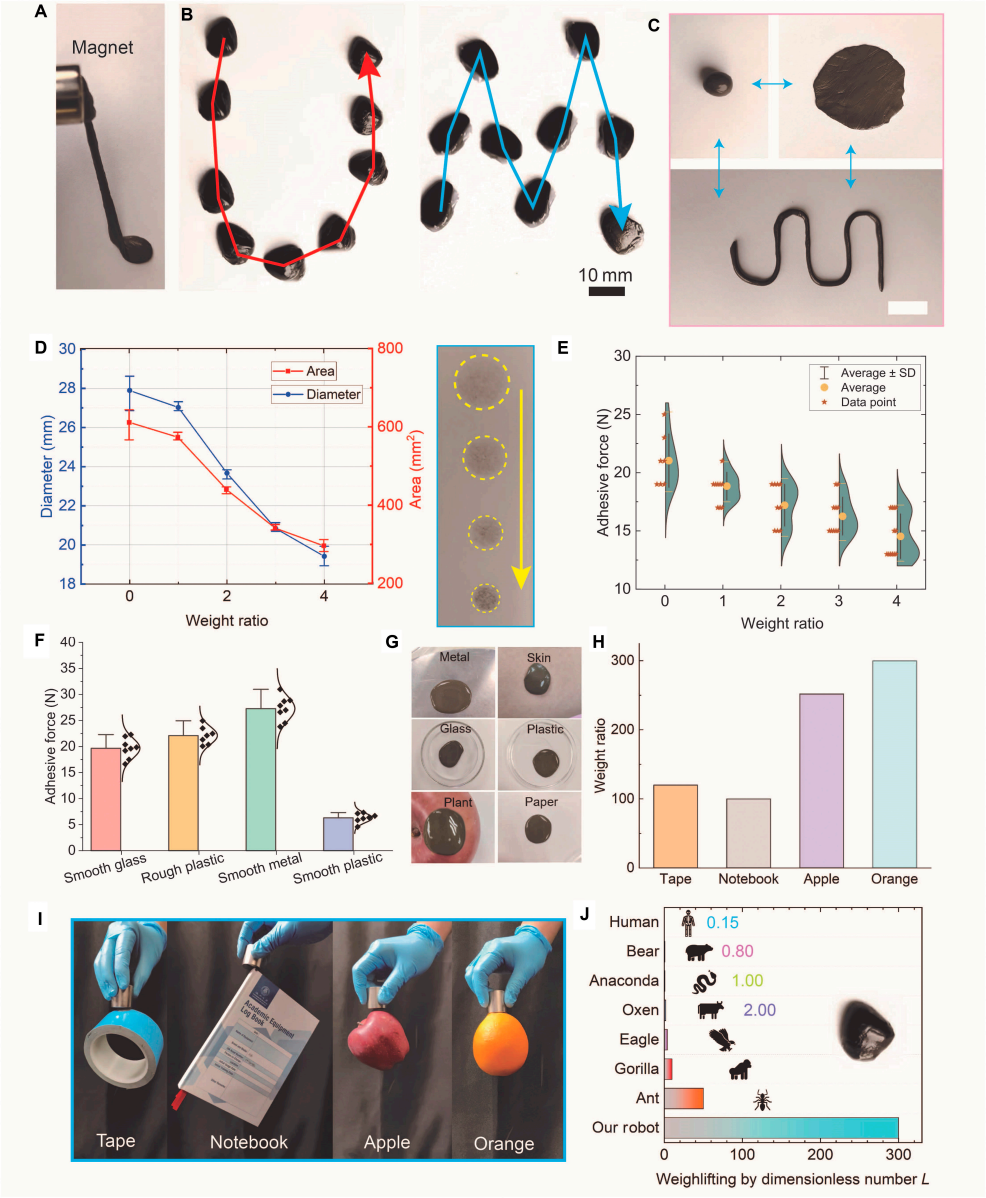
Generality demonstration of magnetic soft robots and the adhesion. (A) Photograph of the proposed magnetic soft robots. (B) Controlled movement of the magnetic miniature soft robot in desired trajectories. (C) The excellent deformability of the magnetic miniature soft robot. (D) Detailed data of shape changes of the magnetic miniature soft robot caused by weight pressures. Insets are photos of the left indentations of soft robots. (E) Detailed data to reveal the relationship between weight ratios and soft robots’ adhesion forces. Weight ratio indicates the ratio of the mass of magnetic particles to the mass of soft non-Newtonian materials, ranging from 0:1 to 4:1. (F) Adhesive forces of the soft robots attached on different surfaces, where the contact area is constant at 9.6 cm^2^. (G) Magnetic soft robots can be attached to various surfaces. (H) The ratios of the weights of the captured objects to robots. (I) The magnetic miniature soft robot grasped various objects much larger than the robot. (J) Comparison of the weightlifting ability of different individuals. The dimensionless number *L* indicates the ratio of the weights of the grasped object to the individual.

The mixture of magnetic particles and soft non-Newtonian materials enables outstanding magnetic actuation and malleability (Fig. 2A and Fig. S1). It can serve as a magnetic miniature soft robot to demonstrate related robotic properties individually (Fig. 2B, Figs. S2 and S3, and Movies S1 and S2). The content of magnetic particles plays a profound role in the produced soft robots, including magnetic properties and initial physical properties (Figs. S4 and S5A and B). To work in cooperation with other medical devices or to achieve flexible deployment, the attractive deformability contributes to adapting to various surfaces (Fig. 2C). A weight of 200 g was applied to the magnetic soft robots, and several indentations were left on the dry paper (Fig. 2D). The increment of the magnetic particles led to smaller shape changes and adhesion areas, as shown in the insets of Fig. 2D. Adhesion is important in deploying the robots on various surfaces. To reveal its detailed adhesive forces, we utilized the proposed robots with different material ratios to test their adhesive forces on smooth glass. The increment of the content of magnetic particles contributes to the reduction of the adhesive ability (Fig. 2E). In fact, the increment of magnetic particles means the reduction of the content of mineral oil, leading to a decrease in fluidic properties. It is more difficult to fill the voids or pores of the substrate surfaces and hold surfaces, achieving interlocking and contracting. Thus, the adhesion is reduced (Section S1). Different adhesion forces on different surfaces are also different. We tested the adhesion force on 4 surfaces. The adhesion mechanisms mainly comprise mechanical interlocking and dispersive adhesion in this work. Hence, the physical properties of attached surfaces are essential to the adhesion. For example, rough surfaces tend to form more mechanical interlocking and provide stronger adhesion, which was well proved by the experimental data (Fig. 2F). In those adhesive force experiments, the contact area is constant at 9.6 cm^2^. The ratio of the mass of non-Newtonian materials to the mass of magnetic particles is 1:1. Also, the deployment scenarios can be further extended to multiple materials, where the soft robot can maintain the adhesion for over 1 h (Fig. 2G). For safety considerations, those soft robots can be easily removed with almost no residue (Fig. S5C). These results proved that the robot had good adhesion and deformability, guaranteeing its effectiveness in diverse scenarios. With the help of these properties, the proposed magnetic miniature soft robots demonstrated attractive performances in manipulating objects much larger than the robot. For example, after a soft robot was attached to designated entities, those objects could be actuated by a permanent magnet (height: 30 mm, diameter: 30 mm) (Fig. S6). A 1-g miniature soft robot could actuate or grasp an orange that was 300 times heavier than the robot (Fig. 2H and I). Compared to natural creatures, the proposed robot had an extremely outstanding force output capacity (Fig. 2J). The proposed design is promising in self-robotic executions and provides splendid magnetic empowerment for current medical devices.

### Spreadable deployment

**Fig. 3.**
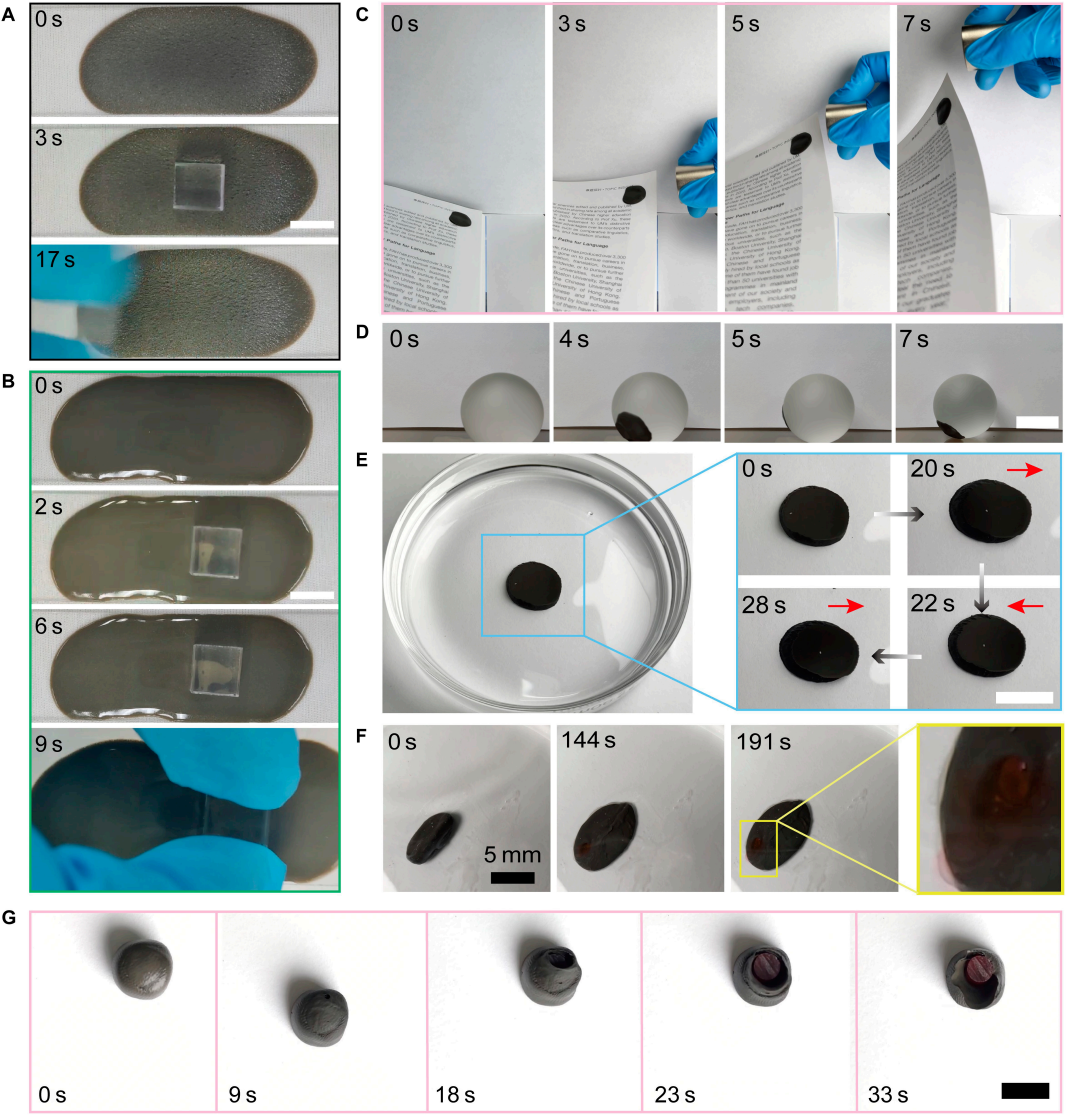
Flexible deployment on various surfaces. (A) Magnetic field-induced adhesion reduction, where the weight is 1 g. (B) Magnetic soft robot surface’s natural adhesion. (C) Flexible deployment enables the actuation of a nonmagnetic object. (D) A thin-film-like soft robot actuated a giant glass ball. (E) A 1-g miniature soft robot shook a more than 200-g weight. (F) On-demand cargo release was realized by deformability. (G) Wireless-controlled on-demand release of an enclosed cargo. All scale bars are 10 mm.

The excellent deformability and adhesion pave a new path to deploy the proposed robots by spreading the robots on various surfaces, where the magnetic empowerment and part of the robotic properties can be maintained. Due to their adhesion, the robots can form a thin film (300 μm or less) attached to the glass surface tightly (Fig. 3A and B). Intriguingly, the properties of the formed surfaces are flexibly modified by applying external magnetic fields. With strong magnetic fields (200-mT level), contained magnetic particles are organized, roughening the outboard surface, and reducing adhesion (Fig. 3A and Fig. S7). Without magnetic fields, the fluidic property of the robot smooths the surface and promotes the effect of adhesion (Fig. 3B). Adhesion modification helps to adapt to different assignments. With the help of sufficient force output ability and adhesion, the magnet could quickly turn a page after attaching less than a 1-g robot to the page (Fig. 3C and Movie S3). Several application experiments were conducted to prove the robots’ advantages further. The magnetic soft robot demonstrated excellent adhesion and deformability and was attached well to a large glass ball. Thus, the glass ball could be wirelessly actuated smoothly (Fig. 3D and Movie S4). In addition, when more than 200 g of weight were applied to the robot, the weight could be easily shaken, proving that the robot had sufficient output forces (Fig. 3E and Movie S5). With their soft structure, many classic designs of similar miniature robots can also be achieved for tasks such as cargo delivery. The proposed robot could enclose designed cargo. When a strong magnetic field (over 6 T/m) was applied, the cargo could be squeezed and expelled inside the robot and released (Fig. 3F, Fig. S8, and Movie S6). This suggests an exciting solution to achieve remotely controlled on-demand release tasks. As a more interesting application, a soft robot can be utilized to design on-demand release capsules, where the soft robot seals the capsule. The sealed part could be reopened after applying strong gradient magnetic fields (more than 6 T/m) to achieve on-demand release (Fig. 3G and Movie S7).

**Fig. 4.**
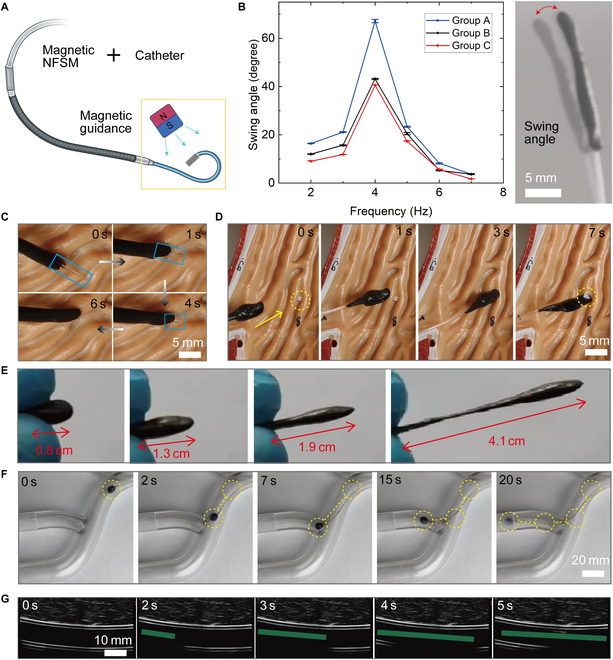
Flexible deployment for functional promotion of traditional manipulation tools. (A) Schematic of attaching a magnetic miniature soft robot to the end of a medical catheter to realize functional enhancement. (B) The magnetic miniature soft robot at the end of a medical catheter responds to the magnetic actuation fields at different frequencies. (C) The magnetic miniature soft robot moves along the catheter. (D) A magnetic miniature soft robot serves as a microgripper to grasp a small object. (E) The controlled elongation of the magnetic miniature soft robot. (F) The magnetic miniature soft robot guides a wire in complex channels. (G) A magnetic miniature soft robot at the end of a medical catheter navigates the channel under the guidance of ultrasound-imaging equipment. NFSM, non-Newtonian fluidic soft materials.

The proposed robots can also be deployed at the end of a traditional medical catheter to realize more functionalities (Fig. 4A). After attaching the soft robot at the end of a catheter, the catheter is capable of magnetic actuation for efficient guidance and execution of a task [5,19–21]. Figure 4B illustrates the responses of a magnetized catheter to external actuation magnetic fields supplied by a rotating magnet, which facilitates detailed applications. The catheter and the proposed magnetic robot can work cooperatively to achieve more intriguing functionalities. For example, the catheter can function as a motion tracking, and the robot moves along it to realize high-efficiency and precise drug delivery (Fig. 4C and Movie S8). Once it arrives at the designated destination, the adhesion of a robot contributes to its ability to grasp small objects by avoiding complex manipulations (Fig. 4D and Movie S9). Although connected at the end of the catheter, the deformability of the robots still demonstrates their splendid advantages. Serving as a kind of end-effector, the outstanding morphological adaptivity helps to navigate more crowded corners (Fig. 4E). In complex channels, the magnetic miniature soft robot is capable of guiding medical wire or other equipment in vivo under the actuation of external magnetic fields (Fig. 4F and Movie S10). All these operations are possibly accessible in vivo with the help of ultrasound equipment (Fig. 4G and Movie S11). In this section, the utilized NdFeB permanent magnet in all experiments has a height of 30 mm and a diameter of 30 mm (Fig. S6). Its flexible deployment paves a new path to fully exploit magnetic soft robots that can potentially improve the performance of the current medical tools.

### On-demand hardening

It is challenging to simultaneously achieve soft robot reconfiguration, deformability, and sufficient mechanical strength, which is even more difficult on a small scale. Herein, we utilized the strong magnetic fields (60 mT at least) to control the magnetic particle distribution status to modify the stiffness and related physical properties. In fact, given the mechanical properties of the non-Newtonian fluid [31–34], sudden stimuli can be applied to activate resistance to external interactions, providing a valuable alternative to designing related miniature soft robots (Fig. S9). Three hardening strategies are provided based on these properties, including external force stimulus, dynamic magnetic field stimulus, and static magnetic field stimulus. Introducing magnetic particles should not reduce the performance of a functional material performance too much in miniature magnetic robot designs [7,35–38].

**Fig. 5.**
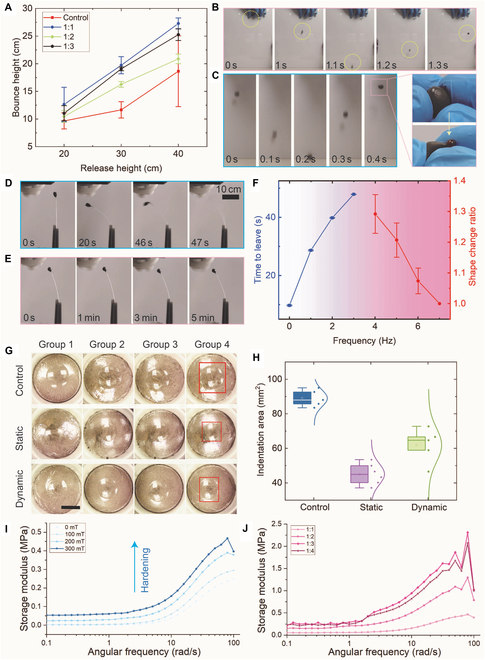
Programmable hardening of magnetic miniature soft robots. (A) The bounce height was induced by dropping the specimen from a release height. The non-Newtonian soft materials and the proposed soft robots with different magnetic contents were tested to compare their mechanical strength and elasticity under external stimuli. (B) Experimental results from the falling and bouncing process for the magnetic miniature soft robot. (C) A magnetic miniature soft robot wrapped and protected liquid cargo during falling and jumping. (D) The magnetic miniature soft robot was actuated by a permanent magnet rotating at a low frequency (3 Hz). (E) The magnetic miniature soft robot was actuated by a permanent magnet rotating at a high frequency (7 Hz). (F) Detailed data of magnetic miniature soft robots under the actuation of a permanent magnet rotating at different frequencies. At low frequencies (<4 Hz), the robot left the end of the catheter. At high frequencies (>4 Hz), actuation promoted mechanical strength. (G) Comparison of indentation under the same pressure and different magnetic actuation fields. Scale bar: 10 mm. (H) Detailed indentation area data revealed the effects of external magnetic fields on the ability of the robot to resist shape changes. (I) Storage modulus of soft robots under different static magnetic fields and angular frequencies, where the ratio is 1:1. (J) Storage modulus of soft robots with different ratios under different angular frequencies, where the static magnetic field is constant at 300 mT.

Different ratios of soft non-Newtonian material weight to magnetic particle weight ranging from 1:1 to 1:3 were tested. After adding magnetic particles, the non-Newtonian fluid could still perform well, whereas the balls of magnetic materials could bounce almost as high as the original materials (Fig. 5A and Movie S12). Detailed experimental results are provided in Fig. 5B. For an intriguing potential application, we utilized the magnetic soft robot to enclose several liquid cargos and protect the cargo when it is dropped (Fig. 5C). This is a promising method for cargo loading in biomedical scenarios, especially when it is vital to protect the cargo. All these results are the hardening enabled by the non-Newtonian fluidic properties, where external force sudden stimuli were applied. The force stimuli can also be mimicked by applying external magnetic fields. In this way, the non-Newtonian fluid property was activated to resist external forces and interactions; namely, it hardened. Rotating magnets generated slow and mild stimuli at low frequencies (<4 Hz). Sudden and intense stimuli were generated by rotating magnets at high frequencies (>4 Hz). In this experiment, the rotating magnet provided a magnetic field with a density of less than 30 mT and a gradient less than 3 T/m. In Fig. 5D, the designed miniature soft robot was attached to the end of a catheter and actuated by a rotating magnet (3 Hz). Within less than 1 min, noticeable shape changes appeared when the magnetic robot finally left the catheter (Movie S13). This illustrates the deformability and morphological adaptivity of the proposed robots. Under such circumstances, magnetic miniature soft robots can almost perform as in previous research. In contrast, the most crucial property, hardening, appeared under actuation by a rotating magnet with high frequency (>4 Hz). During the whole actuation period, the magnetic robot demonstrated only negligible changes in shape and stayed stable at the end of the catheter for over 5 min (Fig. 5E and Movie S14). Numerous experiments were performed to reveal further the relationship between external stimuli and deformability, and detailed data are provided in Fig. 5F. It was evident that the increment of the external rotating magnet’s actuation frequency reduced deformability, where the shape change ratio was almost zero. The most intuitive reflections of the programmable hardening were the shape change resistance and load capacity. To further reveal the differences in hardening procedures, we put the same weight on the magnetic robot to compare the indentation under different conditions (Fig. 5G). Static hardening is achieved by a strong static magnetic field (100-mT level), where the indentation is the smallest (Fig. S7) [30]. The indentations were clearer without actuation by an external changing magnetic field (Fig. 5G). After applying a rotating magnet (20 Hz, 60-mT level magnetic fields) to actuate those magnetic robots, the indentations were well reduced, demonstrating the effectiveness of the proposed hardening strategy (Fig. 5H). It was easy to apply the required magnetic fields. For more detailed hardening data, we utilized a rheometer to measure the storage modulus of magnetic robots with different magnetic fields. Strong magnetic fields contribute to powerful hardening effects, and the increment of magnetic particles also somewhat enhances the hardening (Fig. 5I and J). The normal rheometer cannot fully reveal related data since dynamic magnetic field-induced hardening leads to vibration and force interactions between robots and external environments. Considering the changes in static magnetic fields and angular frequencies, the provided data still can prove the effectiveness of the hardening strategy (Fig. S14). The selection of proper hardening strategies is critical. For static magnetic fields, the soft robot is actuated in one direction, contributing to greater adhesion in the actuated direction, which reduces its locomotion ability. In addition, static magnetic fields will cause unavoidable shape changes in open spaces, which might lead to detachment between the robot and medical tools. The robot is actuated with proper frequencies in dynamic magnetic fields, contributing to considerable vibration. The accompanying force interactions between the robot and external environmental surfaces reduce the adhesion, enabling the robot to move smoothly. In addition, it also led to fewer shape changes so that the attached robot could work stably with medical tools. Thus, it would be possible to achieve programmable hardening to realize more powerful functionalities for designed tasks.

### Hardening-induced functionality enhancement

**Fig. 6.**
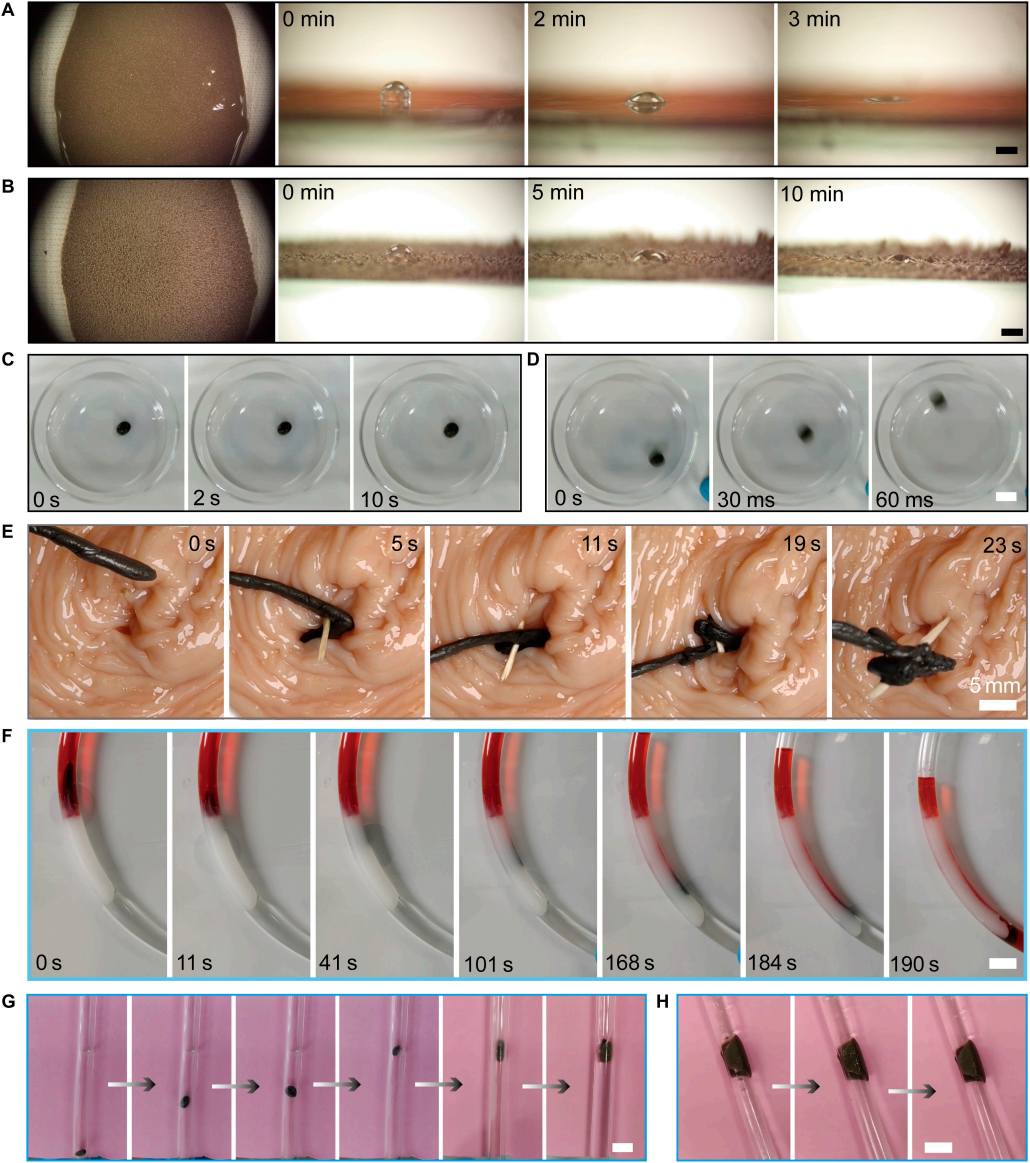
On-demand hardening for various applications. (A) Magnetic soft robots serve as fluidic surfaces, failing to stand a small droplet without magnetic fields. Scale bar: 2 mm. (B) Hardened magnetic soft robot forms relatively rough hard surfaces. Scale bar: 2 mm. (C) Static magnetic actuation of magnetic soft robots contributes to adhesion on the glass surface. (D) Dynamic magnetic actuation of magnetic soft robots contributes to resisting adhesion on the glass surface. Scale bar: 10 mm. (E) A magnetic miniature soft robot pulls out the thorn. (F) A miniature soft robot cleared a blockage made of solid Vaseline. Scale bar: 5 mm. (G) Magnetic soft robot repairs the pipe leak. Scale bar: 3 mm. (H) Magnetic soft robot resists intense water pressures, where the water speed can reach 30 cm/s or more. Scale bar: 3 mm.

Different hardening strategies enable different functionalities with various requirements because different hardening strategies lead to magnetic soft robots’ different accompanied responses. When the proposed robot serves as the adhesive surface, its responses to magnetic fields are intuitive. Without magnetic fields, the formed film is smooth and ultrasoft, where a tiny droplet can leave visible indentations (Fig. 6A). Applying a strong static magnetic field (100-mT level), the formed surfaces are uneven and with small burrs, which is a sign of static hardening (Fig. 6B). Thus, it is accessible to realize flexible modification of targeted surfaces. In addition, the outstanding adhesion of the proposed robots tends to cause undesired adhesion on surfaces, preventing smooth navigation. This problem can also be solved by utilizing hardening strategies. Under these circumstances, static magnetic fields or slow-changing magnetic fields contribute to adhesion promotion (Fig. 6C). By using the dynamic magnetic fields (10 to 20 Hz, fast-changing), the proposed robot can achieve smooth, controlled movement on the glass surface (Fig. 6D). More importantly, as a kind of gripper, the soft robot provides sufficient output forces to remove the thorn inserted into the tissue (Fig. 6E and Movie S15), proving the hardening-induced performance enhancement. Hardening strategies bring lots of stiffness promotion so that several almost impossible assignments to those liquid-based robots can be finished. For potential medical applications, we utilized the sufficient output forces of the robot to clear a model embolism in a constrained channel, where the embolism was made of solid Vaseline (Fig. 6F and Movie S16). The miniature robot succeeded in navigating the solid Vaseline smoothly. Furthermore, the soft robot can demonstrate excellent shape adaptivity and stiffness promotion at the same time. It provides an attractive solution to repair the pipe leak and resist strong water pressure (speed: 30 cm/s or more). Based on those findings, the proposed robots bring novel utilization and deployment strategies for various application scenarios, proving the effectiveness and reliability of such a physical intelligence-inspired design.

## Discussion

In this study, we aimed to design a magnetic miniature soft robot that could quickly achieve programmable hardening and outstanding morphological adaptivity to enrich its functionalities. Swift and programmable hardening was the most important contribution of this study. In addition, we also proposed a deployment strategy to promote current medical device performance. We introduced non-Newtonian fluidic soft materials and mixed them with appropriate magnetic microparticles. When swiftly changing magnetic fields were applied to actuate the magnetic soft robot, its non-Newtonian fluid property was activated to resist external forces and interactions, which was the fundamental basis of programmable hardening. Static magnetic fields can also be utilized to achieve on-demand hardening. The changing frequency of external actuation magnetic fields governed the mechanical properties of the magnetic soft robot. Thus, the designed soft robots could simultaneously exhibit outstanding morphological adaptivity and sufficient mechanical stiffness. Compared with traditional liquid-metal-based robots, the hardening took effect more quickly and flexibly. Indeed, the non-Newtonian materials and magnetic particles were not well designed and processed in advance; this study was only an example to prove the effectiveness of the proposed design strategy. It would be desirable to optimize further the materials adopted in this study to improve their performance substantially.

Another contribution was that the proposed robot could provide sufficient force outputs with the help of stiffness enhancement and outstanding adhesion, even as much as 300 times its weight. Considering its exceptional morphological adaptivity, the powerful output forces were vital and played the best role in the most suitable position, such as navigating constrained corners. In addition, when confronted with irregular objects, its deformability facilitated efficiently catching objects, maximizing the force output effects. Sufficient mechanical stiffness and accompanying force output enhancement could be realized only through quick actuation to activate its non-Newtonian fluid property. Even under such a powerful force output, its soft structure was sufficiently safe to prevent potential physical injuries. The output force ability was strongly dominated by the content of magnetic particles, which should be optimized to promote the magnetic properties to some extent. Biocompatibility is also a crucial concern that is expected to be improved in the future.

Finally, in addition to utilizing the intrinsic properties of the proposed robots, this study also proposed an attractive strategy. That is, through the adhesion of the robots, we can flexibly deploy the robots everywhere to exploit them fully. An excellent example is that the soft robot worked cooperatively with the medical catheter. The robots provided swift magnetic responses for wireless guidance and served as multifunctional end-effectors. Its splendid deformability and force output ability were fully utilized. It provided a great reference to spread magnetic robot applications. Mixing magnetic particles with various functional materials is common in miniature robot research. To overcome the current challenges, fully exploiting the intrinsic properties of materials and having magnetic elements that better activate the functionalities of materials are needed. This study mainly focused on the proposed strategy without considering further material selection and optimization for promoting functionality and addressing biomedical concerns. Hence, achieving more functional designs and practical applications related to the proposed magnetic miniature soft robots would be of great interest to advancing current magnetic robot research.

## Materials and Methods

### Material preparation and experimental setup

The soft non-Newtonian material was produced by 3% mineral oil and 97% carbon silicone gel, demonstrating soft structures and attractive non-Newtonian properties. The adopted magnetic particles were NdFeB microparticles (<5 μm) and 300-mesh Fe-Ni alloy particles (Figs. S10 and S11). Magnetic soft robots were produced by evenly mixing the soft materials and magnetic particles at desired weight ratios (Figs. S12 and S13). The maximum weight ratio of magnetic particles to non-Newtonian materials was 4:1. No additional requirements were needed in the manufacturing procedure. The magnetic soft robots composed of NdFeB are blackish, and those composed of Fe-Ni alloy particles are grayish. They are easy to distinguish in experiments. We also utilized a rheometer to measure the related parameters of the magnetic robots (Fig. S14). A cylindrical NdFeB permanent magnet was used to provide the desired magnetic fields (Fig. S6). To generate rapidly changing magnetic fields, the cylindrical NdFeB permanent magnet was fixed on a motor and rotated at different frequencies (Fig. S15). Adhesion force measurement schematic is provided in Fig. S16. A charge-coupled device camera captured all experimental images.

### Magnetic actuation

The introduction of magnetic particles is the basis of the proposed magnetic actuation ability of the robots. In principle, magnetic objects are subject to magnetic forces and torques under external magnetic fields. NdFeB permanent magnets were utilized during the experiments to provide sufficient magnetic field gradients for magnetic force actuation. The magnetic force can be theoretically calculated as follows.

$$F_{m}=\int_{V_{m}}(M\cdot \nabla )B\;dV_{m}$$ (1)

where *V_m_*, *M*, and *B* represent the volume of the magnetized magnetic soft robots, the magnetization of the magnetic soft robots, and the flux density of the magnetic field, respectively. Through the measured data, it was possible to obtain the theoretical forces. In addition, the adhesion and friction forces between the robot and corresponding substrates were crucial to the wireless control procedure. Solid Vaseline was utilized for lubricating, which was applied on the robot surface when needed. Thus, the uncertainties were severe, and related detailed descriptions were highly sophisticated.

### Robot dynamic hardening analysis and description

To better illustrate the hardening procedure, the magnetic soft robots were regarded as a kind of shear-thickening fluid. It was essentially a dense mixture of mineral oil (<3%), carbon silicone gel, and magnetic microparticles, as shown in related SEM images (Figs. S11 and S13). When the magnetic soft robot was at rest or static, the gaps between those particles were minimal, and the mineral oil could fill the gaps and thoroughly lubricate the particles. Thus, those microparticles inside the magnetic soft robot could move relatively smoothly, demonstrating ultrasoft properties. When applying rotating magnets to actuate those magnetic particles, the gaps between those particles changed. Some of the particles could not be well lubricated because the particles tended to be knocked together. The movement of particles inside the magnetic soft robot was obstructed by solid–solid friction, demonstrating the enhancement of the mechanical stiffness, namely, hardening [31]. Furthermore, the dynamic magnetic actuation contributes to the vibration of the proposed robots, where their force interactions with external environments also spur their non-Newtonian fluidic properties in hardening. For a more theoretical description, we utilized the velocity field *u* to represent the fluid. The velocity field was governed by the incompressible Navier–Stokes equation [39].

∇⋅u=0 (2)

$$\rho \frac{\mathrm{Du}}{\mathrm{Dt}}=-\;\nabla p+\nabla \cdot \left[\lambda \left(x\right)\varepsilon \left(\nabla u+\nabla u^{T}\right)\right]+F_{m}+F_{c}+F_{g}$$ (3)

where *ρ*, *u*, and *p* represent the density, velocity vector, and pressure, respectively. *F_m_*, *F_c_*, and *F_g_* denote the magnetic force, capillary force, and gravity-induced body force, respectively. *λ* = *λ*(*x*) is the viscosity ratio extended to the entire domain, equal to one for the continuous phase. *ε* is the viscosity of the continuous phase, i.e., the environment where we conducted robot experiments. The viscosity of magnetic soft robot *η*(*ϑ*) depends on the state variable *ϑ* within [0, 1], which is a scalar field. *ϑ* denotes the local state of the magnetic soft robot. When *ϑ* = 1, those particles inside the magnetic soft robot are jammed, indicating hardening. We utilize the Vogel–Fulcher type divergence to approximate the severe thickening as follows [33,34].

$$\eta \left(\vartheta \right)=\eta_{0}\text{exp}\left[\frac{\mathrm{A\vartheta}}{1-\vartheta }\right]$$ (4)

where *A* is a dimensionless constant. When the state is at rest, the steady value *ϑ*_∗_ is determined by the local shear stress *γ*. The description is given below.

$$\vartheta_{*}\left(\gamma \right)=\vartheta_{0}\frac{{\left(\gamma /\gamma_{0}\right)}^{2}}{1+{\left(\gamma /\gamma_{0}\right)}^{2}}$$ (5)

where *γ*_0_ is the characteristic stress and *ϑ*_0_ is the limiting value, which represents the state in the stress limit and depends on the detailed properties of the magnetic soft robot, such as the packing fraction of the dispersed granules.

### Robot static hardening analysis and description

For the static magnetic field-induced hardening, when applying static magnetic fields (>100 mT), extra hardening details exist, as shown in Fig. S7. After applying strong magnetic fields, magnetic particles are all magnetized, which can be regarded as a small magnet. Those small magnets are organized as chain-like structures, which is the bone structure of proposed soft robots, promoting stiffness. When those magnetic particles are magnetized and organized, subject to strong external magnetic fields, particles inside the mineral oils tend to be locked since the oil cannot well lubricate those particles. This also contributes to the hardening procedure.

### Data analysis and statics

The results are presented as the means ± SD. Without other specifications, all experiments were repeated at least 3 times independently.

## Data Availability

All data generated or analyzed for this paper are included in the published article, its methods, and its Supplementary Materials. Original videos and sensor data are available from the corresponding author on reasonable request.
