# Supplementary material for: Spreadable Magnetic Soft Robots with On-Demand Hardening
Source: Research (Wash D C). 2023 Nov 29;6:0262. doi: 10.34133/research.0262 (PMC10687580; doi:10.34133/research.0262)
Supplement: Supplementary 1 — Figs. S1 to S16 Movies S1 to S16 [file research.0262.f1.zip › Research-sup-R1.pdf]

Supporting Information

Spreadable magnetic soft robots with on-demand hardening

*Zichen Xu, Yuanhe Chen, and Qingsong Xu**

The PDF file includes:

Supplementary Text

Figures:

Fig. S1. Relationship between the static placement time and contact angle of the soft non-Newtonian materials (without magnetic particles) on a smooth plastic substrate. Inset photographs show the experimental results for the material at different times.

Fig. S2. The morphological adaptivity of the magnetic miniature soft robot facilitated passing through the complex channel.

Fig. S3. The deformability of the magnetic miniature soft robot enabled flexible manipulation tasks.

Fig. S4. Magnetic hysteresis loops of magnetic soft robots with different amounts of magnetic particles. The weight ratios of the magnetic soft robot to magnetic particles were 1:1, 1:2, and 1:3.

Fig. S5. a) Phase diagram of the deformability of magnetic miniature soft robots passing through crowded channels of different widths. η denotes the mass ratio of the magnetic particles to soft non-Newtonian materials. The red region indicates that the robot could pass through the channel depicted by the image in the red box. The purple region indicates that the robot could

not pass through the channel, as shown by the image in the purple box. **b)** The time versus weight ratio of the robots passing through the channels of different widths.

Fig. S6. COMSOL simulation results of magnetic field distribution for the adopted NdFeB permanent magnet.

Fig. S7. Schematic of the particle distribution inside the soft robots. The grey ball indicates the normal particles, and the other one denotes the magnetic particles. After applying strong magnetic fields, magnetic particles are all magnetized, which can be regarded as a small magnet. These small magnets are organized as chain-like structures, which is the bone-structure of proposed soft robots, promoting stiffness. In addition, the surfaces is roughing, reducing the adhesion.

Fig. S8. COMSOL simulation results of the stress distribution for the actuated soft robot. *d* denotes the distance between the magnet and soft robot.

Fig. S9. Responses of the non-Newtonian materials to various external stimuli. Gentle and slow stimulation changed its shape. The materials could resist sudden blows to some extent, namely, hardening. The constant force works well to be stretched.

Fig. S10. Magnetic hysteresis loops of the NdFeB particles.

Fig. S11. SEM image of the adopted NdFeB microparticles.

Fig. S12. Schematic of the fabrication of a magnetic soft material made of a mixture of non-Newtonian materials and magnetic particles.

Fig. S13. SEM images of the proposed magnetic soft robots.

Fig. S14. Experimental setup of a rotating permanent magnet platform to provide magnetic actuation fields.

Other Supplementary Material for this manuscript includes the following:

Movie S1. The magnetic miniature soft robot passes through a complex channel via morphological adaptivity.

Movie S2. The magnetic miniature soft robot realizes flexible manipulation tasks via excellent deformability.

Movie S3. The flexible deployment enables the actuation of non-magnetic objects.

Movie S4. A thin film-like soft robot enables the actuation of a big glass ball.

Movie S5. One-gram miniature soft robot shakes a weight of more than 200 grams.

Movie S6. The magnetic soft robot induces on-demand cargo release by deformability.

Movie S7. Wirelessly controlled on-demand release of the enclosed cargo.

Movie S8. The magnetic miniature soft robot moves along a catheter.

Movie S9. The magnetic miniature soft robot acts as a microgripper to grasp small objects.

Movie S10. The magnetic miniature soft robot guides a wire in complex channels.

Movie S11. The magnetic miniature soft robot at the end of a medical catheter navigates a channel under the guidance of ultrasound-imaging equipment.

Movie S12. The bouncing ability of the magnetic soft robot.

Movie S13. The magnetic miniature soft robot is actuated by a rotating permanent magnet at low frequency.

Movie S14. The magnetic miniature soft robot is actuated by a rotating permanent magnet at high frequency.

Movie S15. The magnetic miniature soft robot pulls out a thorn.

Movie S16. The miniature soft robot clears the blockage made of solid Vaseline.

Section S1. Adhesion of soft robots.

For the theoretical analysis and description of the adhesion behaviors, the adhesion mechanisms are mainly composed of mechanical interlocking and dispersive adhesion (1). For mechanical interlocking, the proposed soft robots demonstrate ultrasoft structures and part fluidic properties. This enables the soft robots to fill the voids or pores of the substrate surfaces and hold surfaces, achieving interlocking. As for the dispersive adhesion, it attributes attractive forces between two materials to intermolecular interactions between molecules of each material (2,3). It is essentially a kind of weak interaction that occurs when molecules are close together. These interactions include London dispersion forces, Keesom forces, Debye forces, and hydrogen bonds. These interactions are weak individually, but they can have strong effects when they work together. In our work, the schematic is presented in the following Figure 1. The contact area is undoubtedly significant to the adhesion. A larger contact area leads to more mechanical interlocking, Van der Waals interactions, and related intermolecular interactions between molecules of each material. During experiments, when the soft robot is not well pressed on the designated surface, it can only provide relatively small adhesion. When it is well pressed, namely, in a larger contact area, the adhesion is much stronger. High temperature contributes to the lubrication the mineral oil provides, where the robot will be softer. The enhancement of shape changes reduces the adhesion.

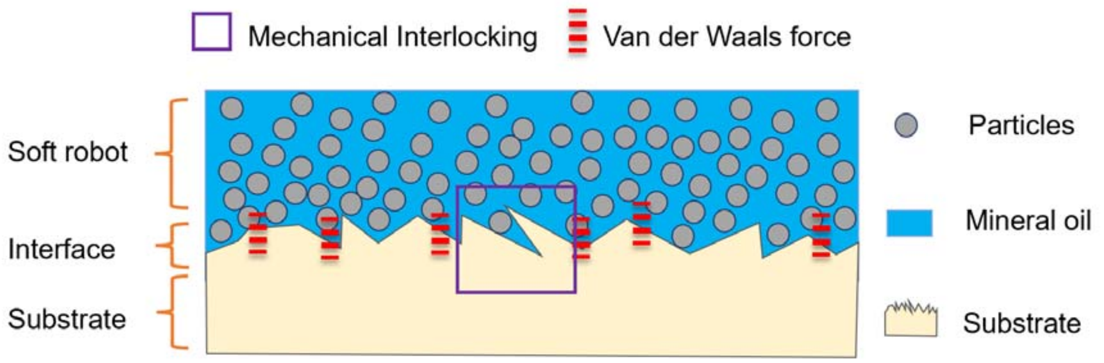

Figure 1. Schematic of soft robots' adhesion mechanism.

1. Kinloch, A.J. (1987). Mechanisms of adhesion. In: Adhesion and Adhesives. Springer, Dordrecht.
2. Wake W C. Theories of adhesion and uses of adhesives: a review [J]. Polymer, 1978, 19(3): 291-308.
3. Fundamentals of adhesion [M]. Springer Science & Business Media, 2013.

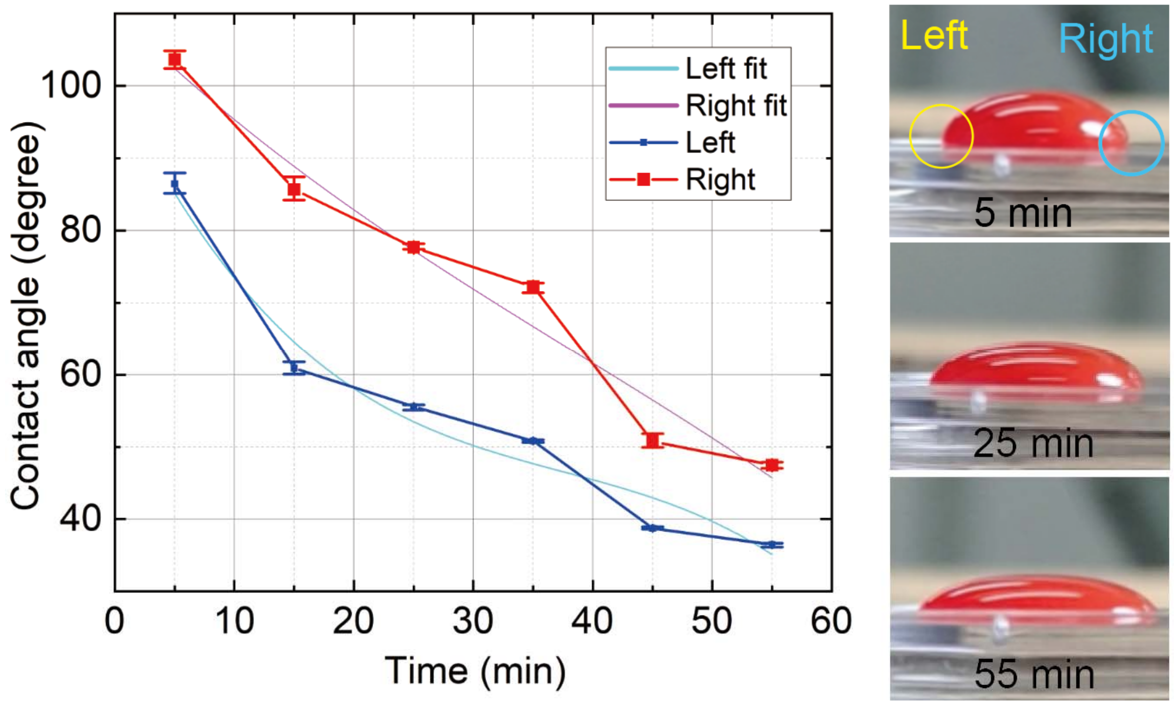

Fig. S1. Relationship between the static placement time and contact angle of the soft non-Newtonian materials (without magnetic particles) on a smooth plastic substrate. Inset photographs show the experimental results for the material at different times.

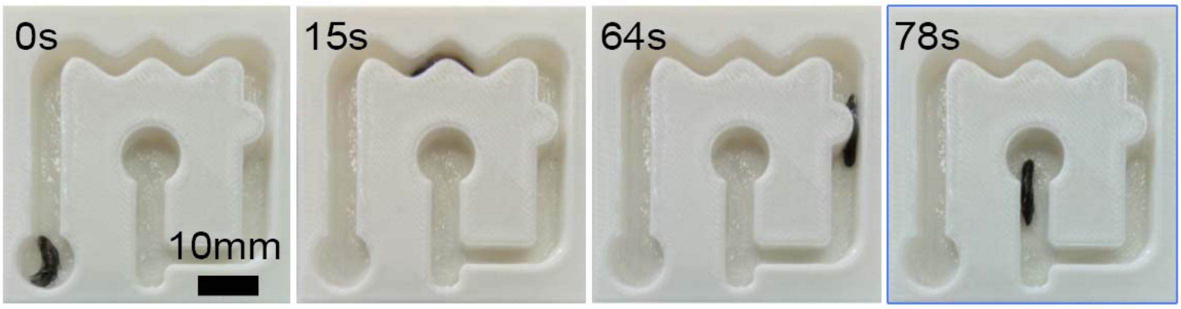

Fig. S2. The morphological adaptivity of the magnetic miniature soft robot facilitated passing through the complex channel.

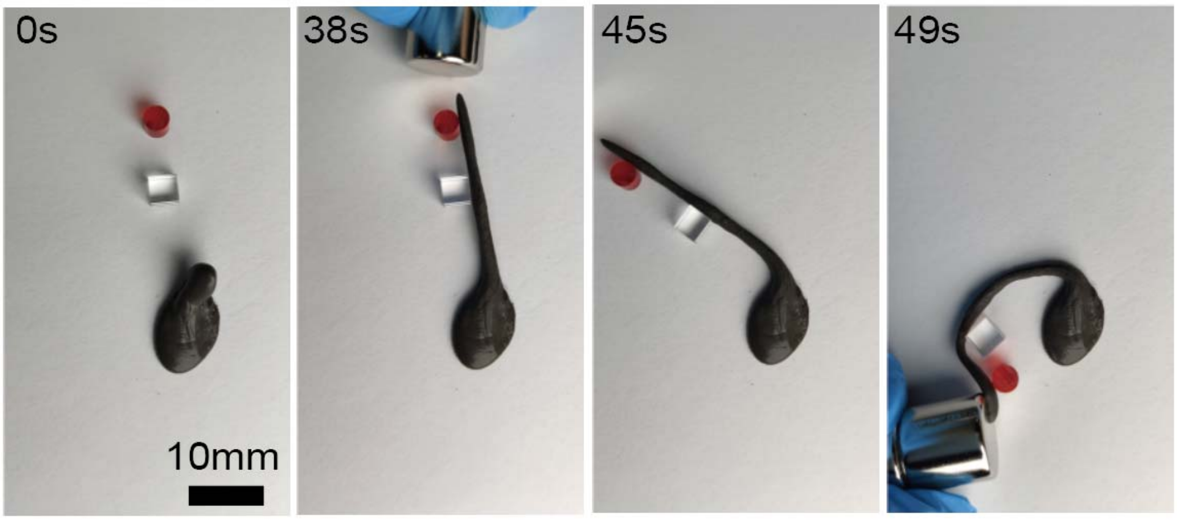

Fig. S3. The deformability of the magnetic miniature soft robot enabled flexible manipulation tasks.

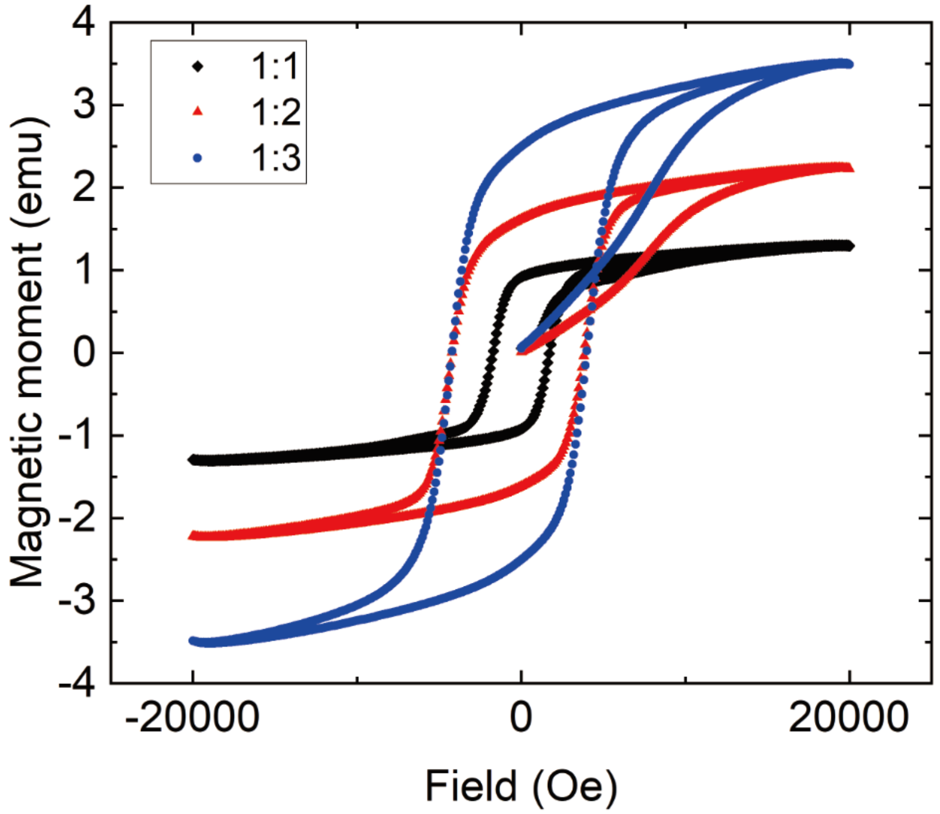

Fig. S4. Magnetic hysteresis loops of magnetic soft robots with different amounts of magnetic particles. The weight ratios of the magnetic soft robot to magnetic particles were 1:1, 1:2, and 1:3.

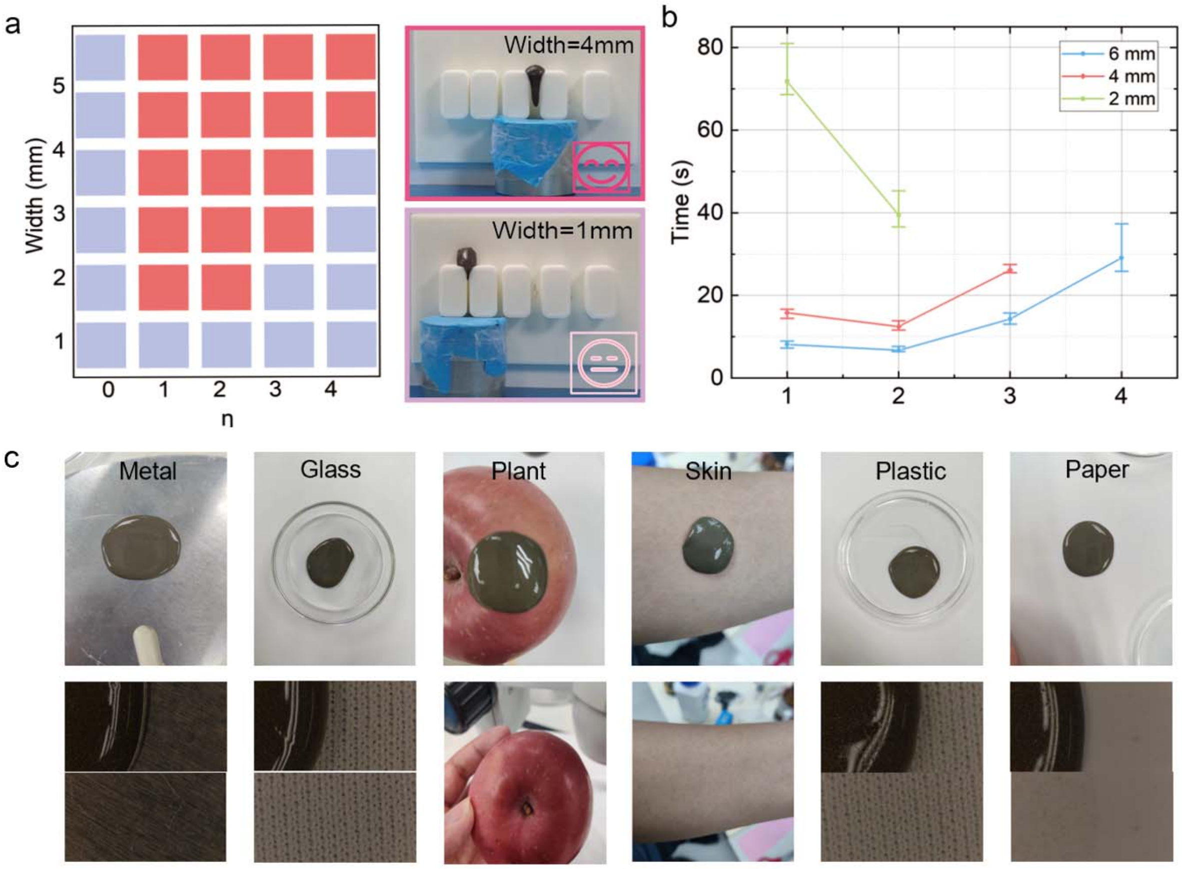

Fig. S5. a) Phase diagram of the deformability of magnetic miniature soft robots passing through crowded channels of different widths. η denotes the mass ratio of the magnetic particles to soft non-Newtonian materials. The red region indicates that the robot could pass through the channel depicted by the image in the red box. The purple area means the robot could not pass through the channel, as shown by the image in the purple box. **b)** The time versus weight ratio of the robots passing through the channels of different widths. **c**) Experimental images about the soft robots' attachment and detachment.

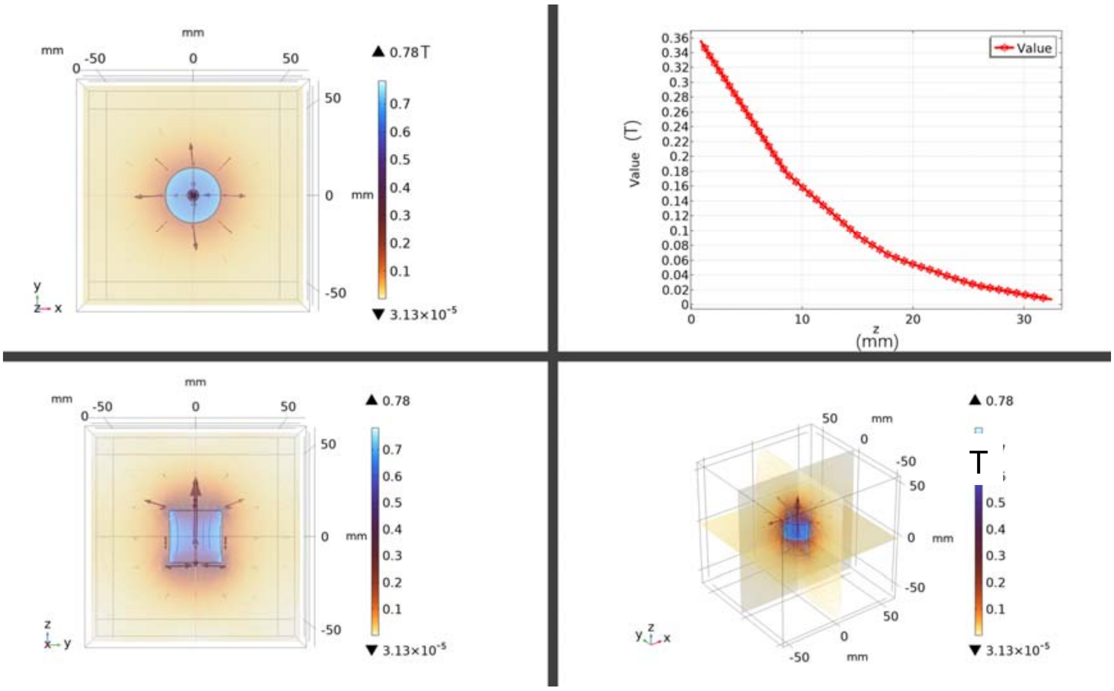

Fig. S6. COMSOL simulation results of magnetic field distribution for the adopted NdFeB permanent magnet.

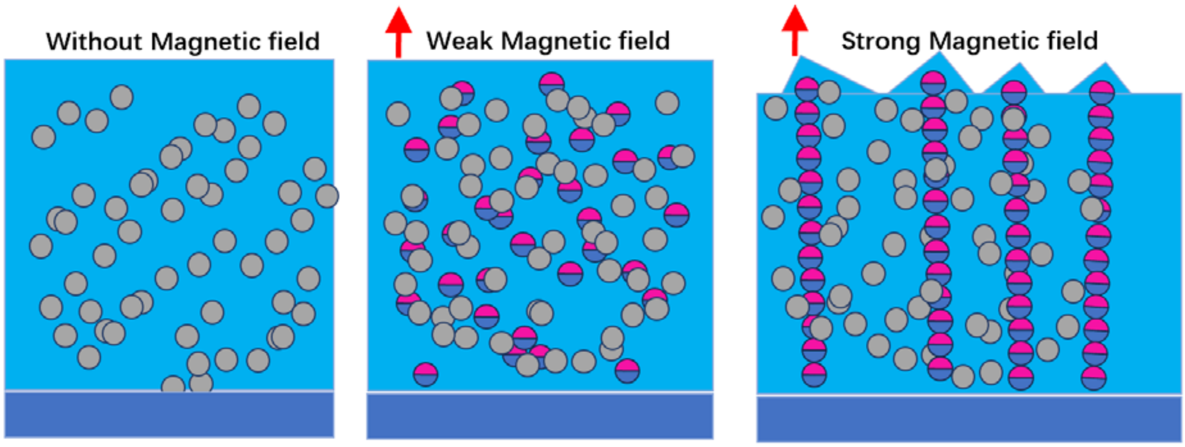

Fig. S7. Schematic of the particle distribution inside the soft robots. The grey ball indicates the normal particles, and the other one denotes the magnetic particles. After applying strong magnetic fields, magnetic particles are all magnetized, which can be regarded as a small magnet. Those small magnets are organized as chain-like structures, which is the bone-structure of proposed soft robots, promoting stiffness. In addition, the surfaces is roughing, reducing the adhesion.

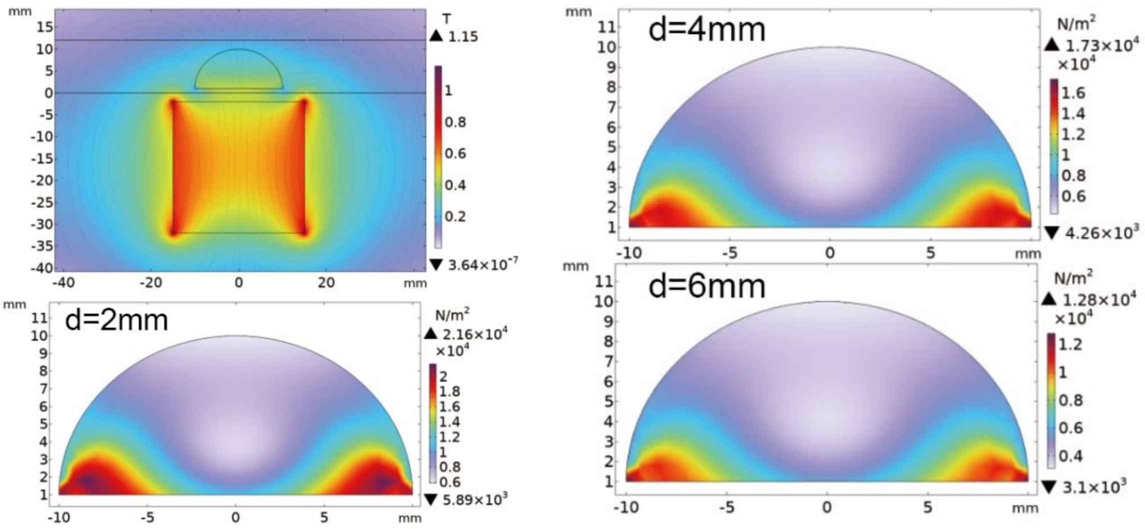

Fig. S8. COMSOL simulation results of the stress distribution for the actuated soft robot. *d* denotes the distance between the magnet and the soft robot.

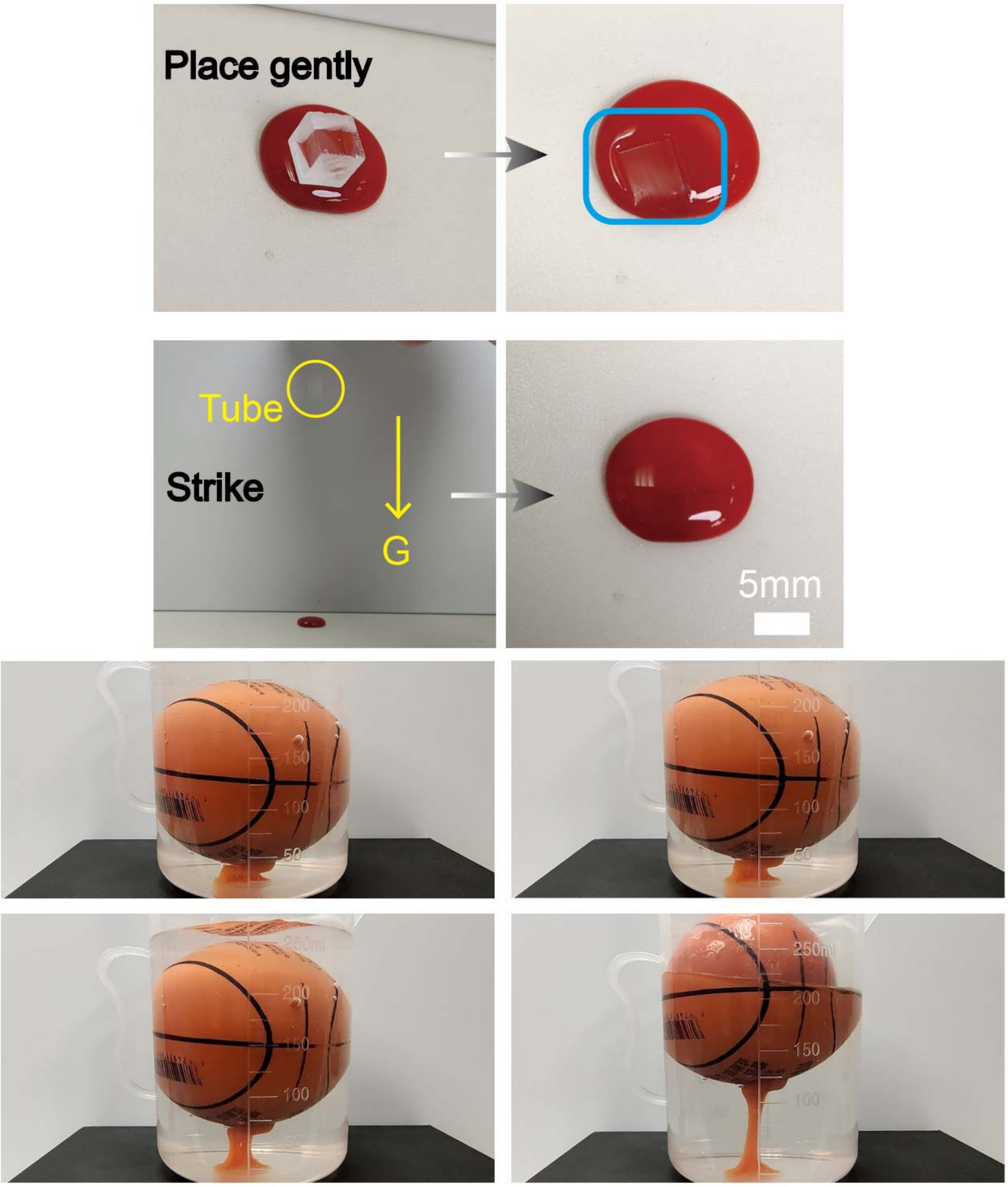

Fig. S9. Responses of the non-Newtonian materials to various external stimuli. Gentle and slow stimulation changed its shape. The materials could resist sudden blows to some extent, namely, hardening. The constant force works well to be stretched.

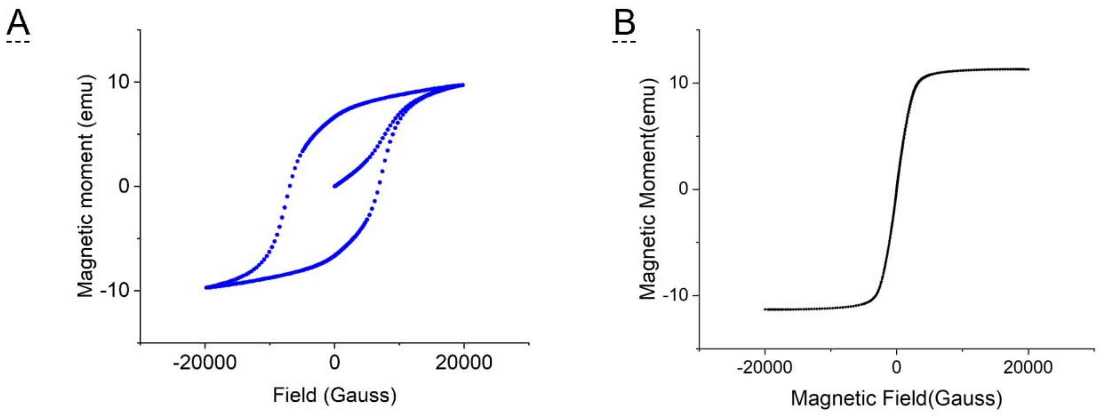

Fig. S10. (A) Magnetic hysteresis loop of the NdFeB particles. (B) Magnetic hysteresis loop of the Fe-Ni alloy particles.

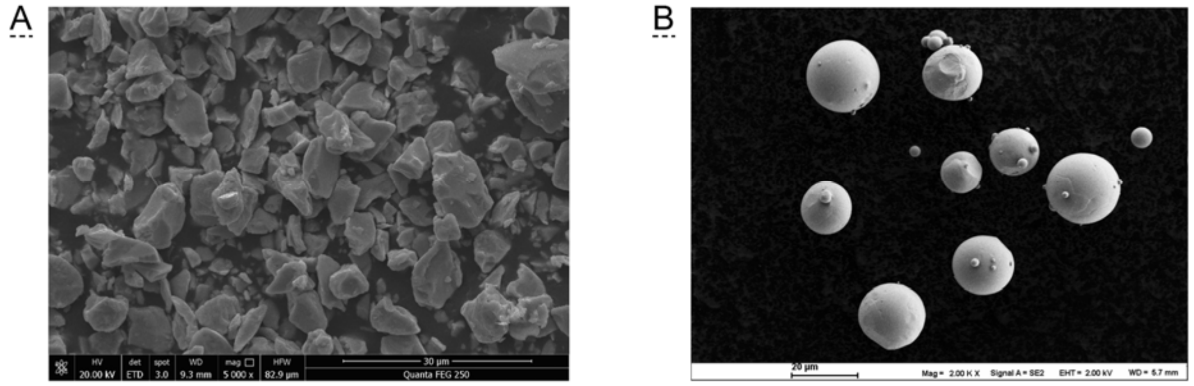

Fig. S11. (A) SEM image of the adopted NdFeB microparticles. (B) SEM image of the adopted Ni-Fe alloy microparticles.

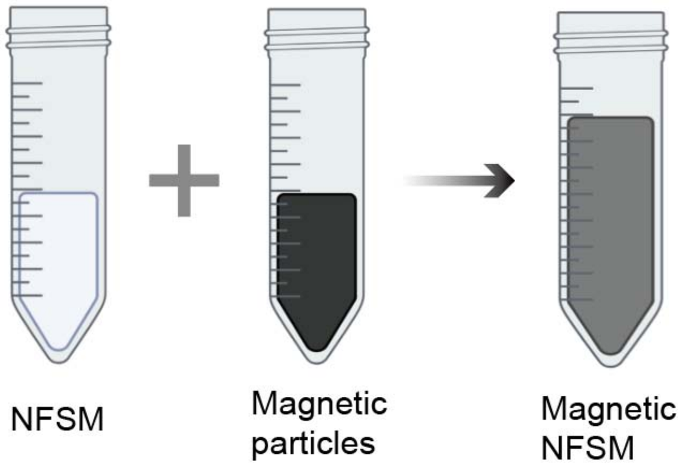

Fig. S12. Schematic of the fabrication of the magnetic soft material made of a mixture of non-Newtonian materials and magnetic particles.

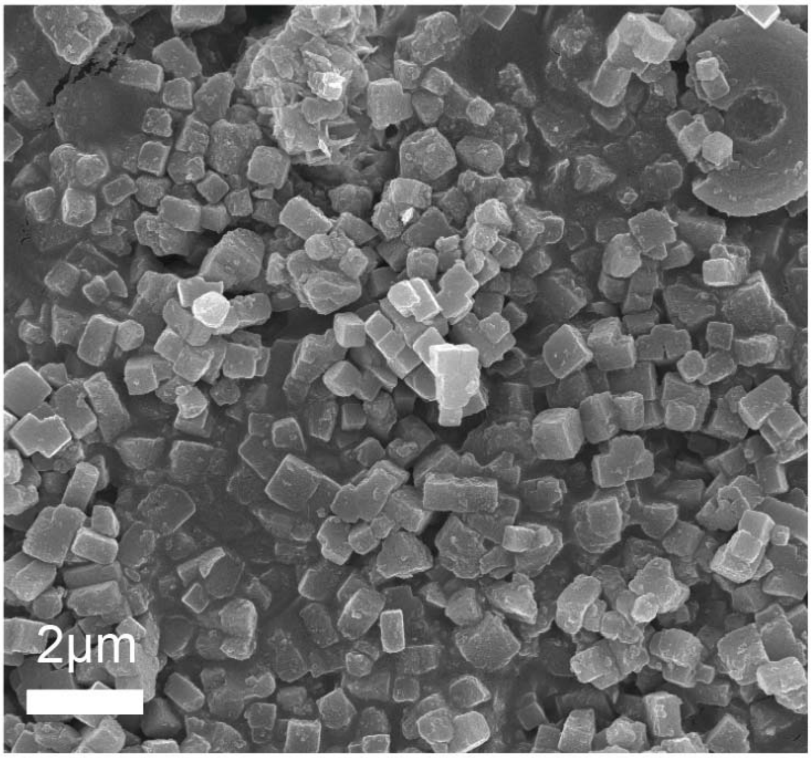

Fig. S13. SEM images of the proposed magnetic soft robots.

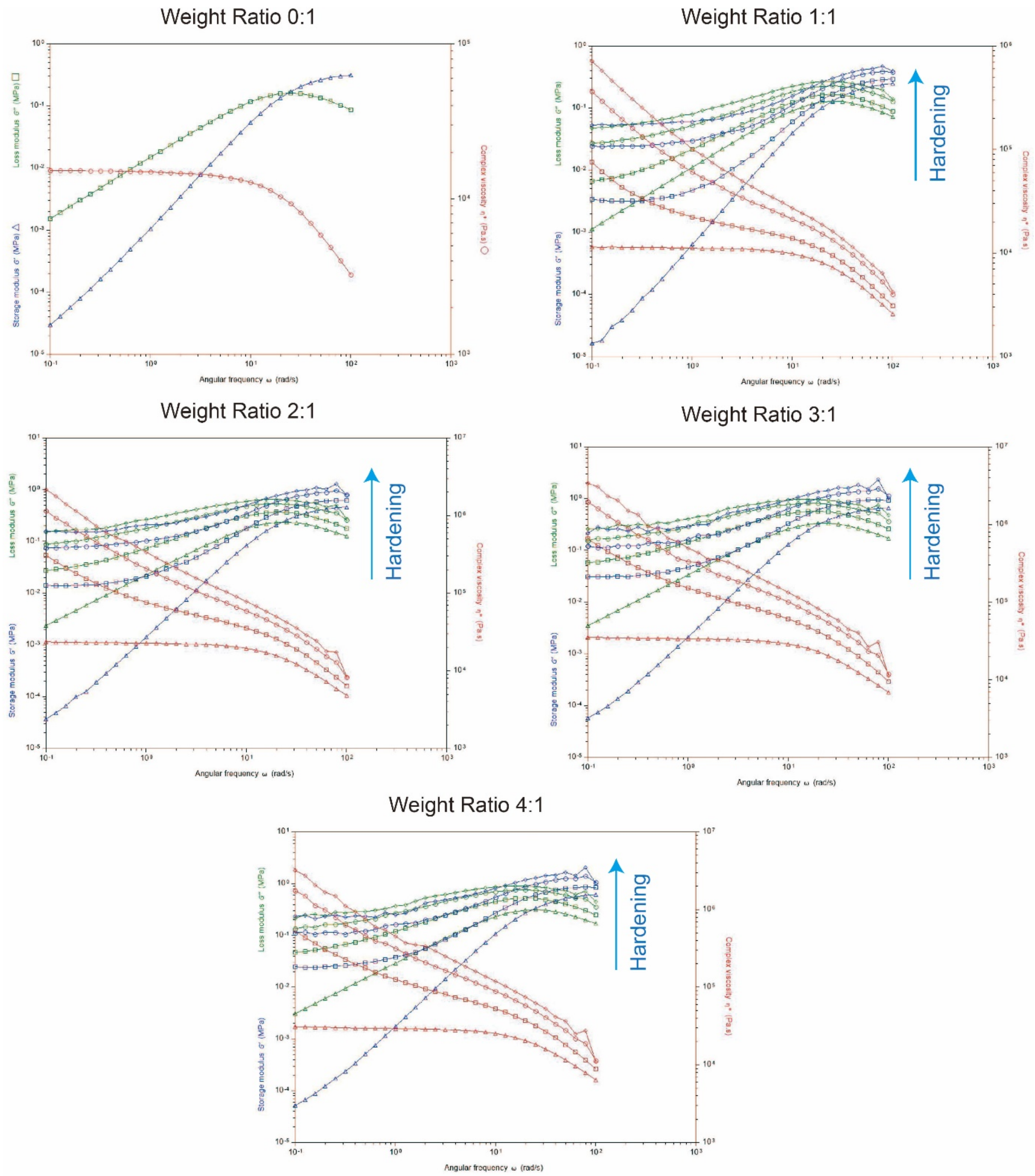

Fig. S14. Rheometer measure of magnetic robots with different weight ratios. The blue arrow indicates the increment of static magnetic fields, including 0 mT, 100 mT, 200 mT, and 300 mT. The detailed data can sufficiently prove the hardening properties enabled by external magnetic fields.

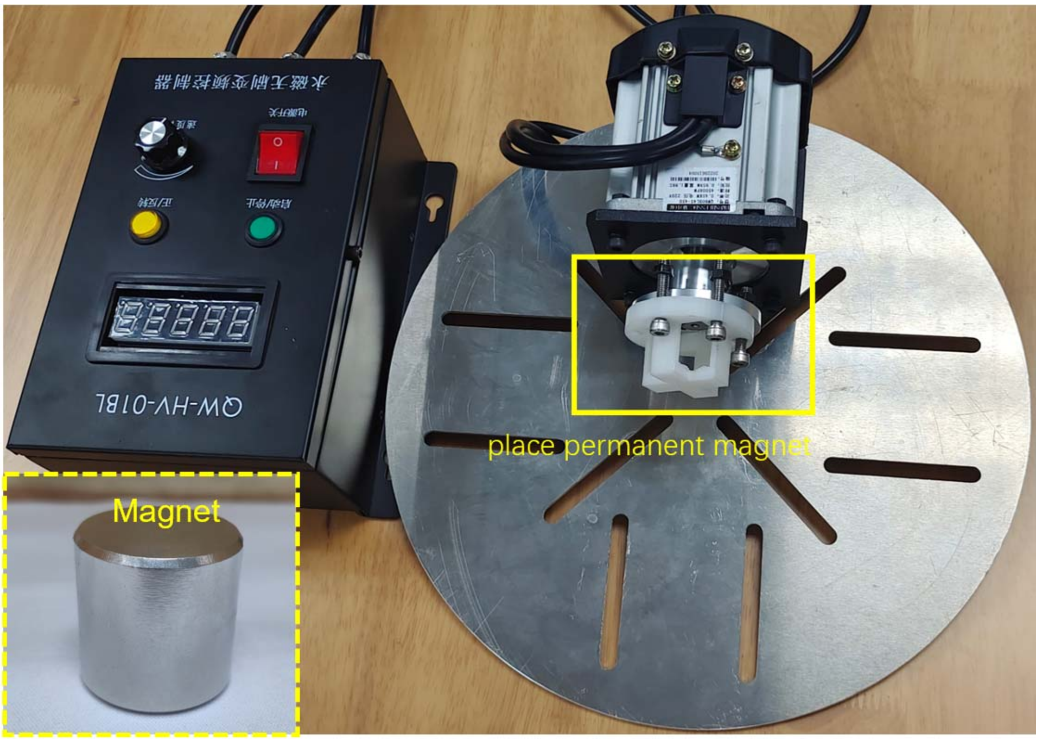

Fig. S15. Experimental setup of a rotating permanent magnet platform to provide magnetic actuation fields. The magnet has a height of 30 mm and a diameter of 30 mm.

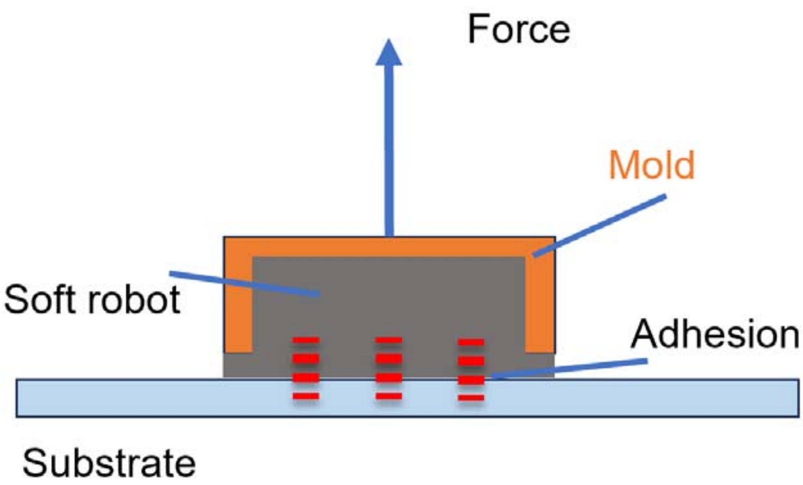

Fig. S16. Schematic of adhesion measurement.
